# Estimation of physiologic ability and surgical stress (E-PASS) scoring system could provide preoperative advice on whether to undergo laparoscopic surgery for colorectal cancer patients with a high physiological risk

**DOI:** 10.1097/MD.0000000000007772

**Published:** 2017-08-18

**Authors:** Ao Zhang, Tingting Liu, Kaiyuan Zheng, Ningbo Liu, Fei Huang, Weidong Li, Tong Liu, Weihua Fu

**Affiliations:** a Department of General Surgery, Tianjin Medical University General Hospital, Tianjin; b Department of General Surgery, Handan First Hospital, Handan, China.

**Keywords:** colorectal cancer, estimation of physiologic ability and surgical stress (E-PASS), laparoscopic surgery, postoperative morbidity, preoperative risk score (PRS)

## Abstract

Laparoscopic colorectal surgery had been widely used for colorectal cancer patient and showed a favorable outcome on the postoperative morbidity rate. We attempted to evaluate physiological status of patients by mean of Estimation of physiologic ability and surgical stress (E-PASS) system and to analyze the difference variation of postoperative morbidity rate of open and laparoscopic colorectal cancer surgery in patients with different physiological status.

In total 550 colorectal cancer patients who underwent surgery treatment were included. E-PASS and some conventional scoring systems were reviewed to examine their mortality prediction ability. The preoperative risk score (PRS) in the E-PASS system was used to evaluate the physiological status of patients. The difference of postoperative morbidity rate between open and laparoscopic colorectal cancer surgeries was analyzed respectively in patients with different physiological status.

E-PASS had better prediction ability than other conventional scoring systems in colorectal cancer surgeries. Postoperative morbidities were developed in 143 patients. The parameters in the E-PASS system had positive correlations with postoperative morbidity. The overall postoperative morbidity rate of laparoscopic surgeries was lower than open surgeries (19.61% and 28.46%), but the postoperative morbidity rate of laparoscopic surgeries increased more significantly than in open surgery as PRS increased. When PRS was more than 0.7, the postoperative morbidity rate of laparoscopic surgeries would exceed the postoperative morbidity rate of open surgeries.

The E-PASS system was capable to evaluate the physiological and surgical risk of colorectal cancer surgery. PRS could assist preoperative decision-making on the surgical method. Colorectal cancer patients who were assessed with a low physiological risk by PRS would be safe to undergo laparoscopic surgery. On the contrary, surgeons should make decisions prudently on the operation method for patient with a high physiological risk.

## 1 Introduction

Surgical resection is an important treatment for colorectal cancer. Radical resections with complete mesocolic excision (CME) or total mesorectal excision (TME) had become standard procedures and were believed to be able to improve outcomes of colonrectal cancer.^[1,2]^ For those with more advanced disease which cannot be radical resected, surgical intervention still can be an alternative to treat or prevent complications such as ileus, hemorrhage, and so on.^[3]^ Many scoring systems were developed to evaluate the risk of postoperative mortality and morbidity. Physiological and Operative Severity Score for enUmeration of Mortality and morbidity (POSSUM) is a representative one and has multiple modified versions, such as Portsmouth POSSUM (P-POSSUM) and colorectal specialized Cr-POSSUM.^[4,5]^ Tekkis et al^[6,7]^ developed Cr-POSSUM and Association of Coloproctology of Great Britain and Ireland (ACPGBI) system in 2003 and 2004, which were modified to evaluate the risk of mortality of colorectal cancer surgery and had better prediction abilities than POSSUM and P-POSSUM.^[8]^ However, as the surgical techniques developed, the transformations of surgical procedures made the scoring systems mentioned above have more limitations. The study of Law et al^[9]^ showed that POSSUM, P-POSSUM, and Cr-POSSUM had excessively overrated the mortality in laparoscopic colorectal surgery. Estimation of physiologic ability and surgical stress (E-PASS) system is a more recent predicting system developed by Haga et al.^[10]^ According to his following examination studies, E-PASS was reported to have a good death predicting ability in various surgeries including colorectal cancer surgery.^[11–13]^

As the minimal invasive techniques become universal and new techniques emerge, more approaches become optional. The laparoscopic technique is widely accepted and has been applied in colorectal surgeries. Many studies gave an affirmative conclusion on laparoscopic surgery, results of which showed better postoperative outcomes than open surgeries.^[14–16]^ However, colorectal surgeries are still traumatic to some extent. Besides, a research reported that laparoscopic rectal surgery did not show advantage on postoperative morbidity in elderly.^[17]^ So we still doubt whether laparoscopic colorectal surgery is suitable for patients with high physiological risk. Scoring systems are ideal options, which are able to assess physiological status of patients and/or assess surgical risk of procedures. We attempted to use parameters in E-PASS to assess the physiological status of patients and analyze the difference of postoperative morbidity rate between open and laparoscopic colorectal cancer surgeries in patients with different physiological statuses.

## 2 Methods

### 2.1 Patients

All colorectal cancer patients who underwent surgical treatment at Tianjin Medical University General Hospital from 2010 to 2015 were reviewed. Cases with missing data were excluded. Cases of laparoscopic surgery converted to open surgery were categorized into open surgery. Postoperative morbidity was classified according to the Clavien–Dindo Classification and defined as complication which is need medical intervention.^[18]^ Diagnosis of postoperative morbidity was supported by clinical manifestation, imaging or laboratory findings.

### 2.2 Scoring systems

Data of age, cardiac function, respiratory function, systolic blood pressure, pulse, Glasgow coma score, hemoglobin, white cell count, sodium, potassium, urea, electrocardiogram, operative severity, number of procedures, blood loss, peritoneal soiling, cancer status, and operative urgency were collected to review the POSSUM-based systems. The detailed calculation process was described in previous reports.^[4,5,7]^

Data of age, American Society of Anesthesiology (ASA) grade, Dukes staging of cancer, operative urgency, and cancer resection status was collected to review the ACPGBI system. Detailed calculation process was described in the previous report.^[6]^

The E-PASS consists of physiological part named “preoperative risk score” and surgical part named “surgical risk score” (SSS). The PRS consists of age, cardiac disease, pulmonary function, diabetes mellitus, performance status, and ASA grade. The SSS consists of ratio between blood loss and weight, operation duration, and extent of skin incision. PRS and SSS were inputted into a formula to obtain “comprehensive risk score” (CRS) which is used to predict postoperative mortality. The detailed calculation process was described in the previous report.^[10]^

### 2.3 Statistical analysis

The quantitative values were given as mean ± SE. The Mann–Whitney *U* test was used to compare measurement data. The Fisher exact and chi-square tests were used to compare counting data. Correlation analyses between continuous variables and rank variables were conducted using the Spearman correlation test. Receiver operating characteristic (ROC) curves were generated and the area under ROC curves (AUC) was used to assess the discrimination ability of prediction models. The higher AUC value represented better discrimination ability. Calibration ability of prediction models was assessed by the Hosmer–Lemeshow statistic. A more significant result of Hosmer–Lemeshow test indicated that a model was more lack of fit. Multivariate logistic analysis was used to determine factors affecting the presence of morbidity. The *P* value less than 0.05 were accepted as significant. Statistics analysis was performed using SPSS version 20.0 software (SPSS Inc., Chicago, IL).

## 3 Results

### 3.1 General information

A total of 550 cases were included eventually. However, 375 (68.18%) patients developed colon cancer, and 175 (31.82%) developed rectal cancer. There were 281 (51.09%) males and 269 (48.91%) females. Age ranged from 27 to 95 years, with a median age of 65 years. There were 6 patients died postoperatively, and all of them died within 30 days in hospital. The morbidity rate was 26.0% and the mortality rate was 1.09%. Other details were listed in Table 1. All death occurred during hospitalization and within 30 days. Two died of pulmonary infection, 2 died of anastomotic leakage, 1 died of pancreatic fistula, and 1 died of abdominal hemorrhage. All cases of death were cases of open surgery.

**Table 1:**
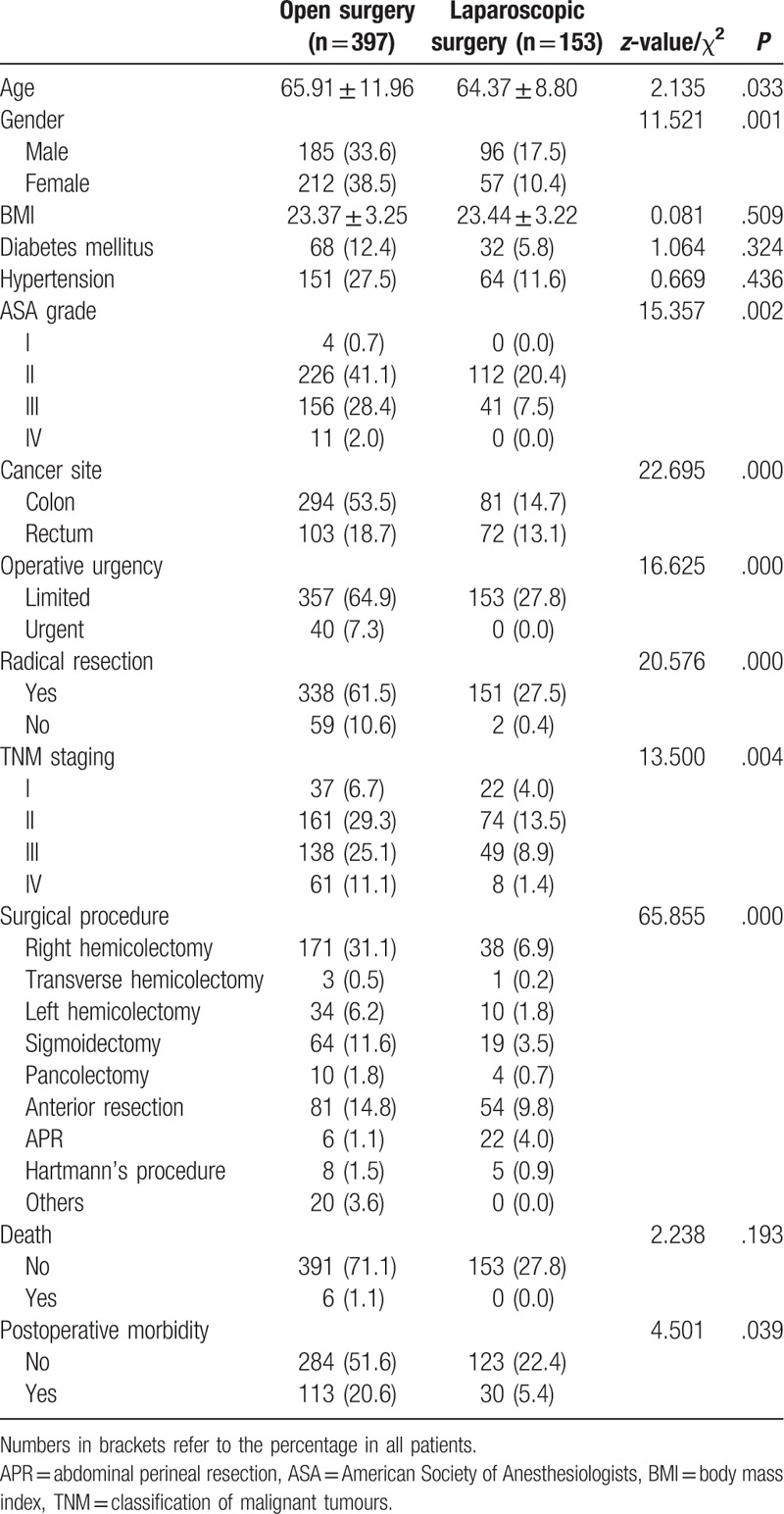
General information.

### 3.2 Efficiency of E-PASS, POSSUM-based system, and ACPGBI in predicting mortality

**Figure 1:**
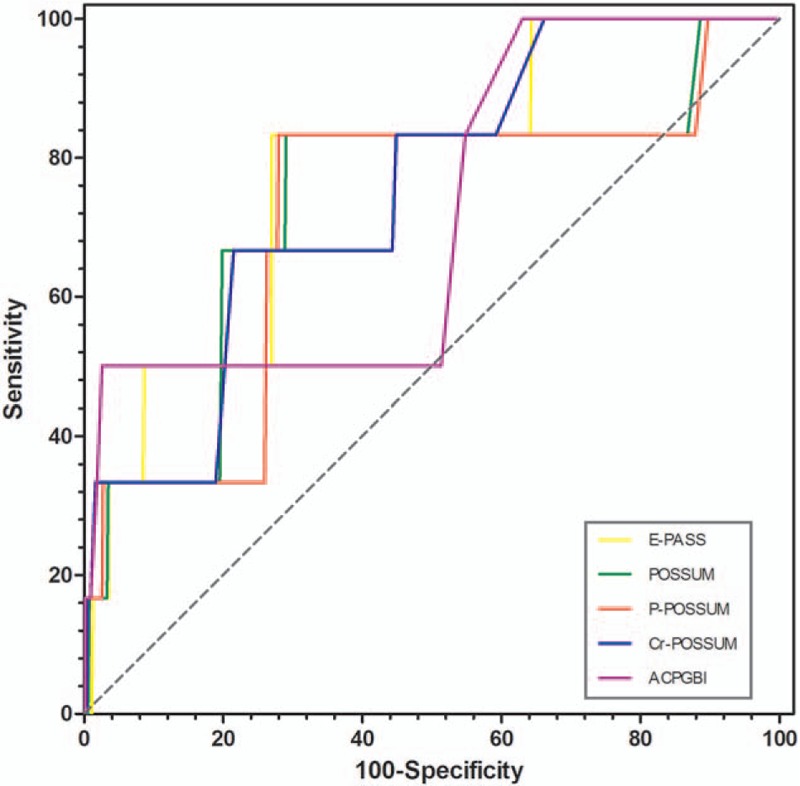
ROCs of POSSUM-based system, ACPGBI system, and E-PASS system. ACPGBI = Association of Coloproctology of Great Britain and Ireland, E-PASS = estimation of physiologic ability and surgical stress, POSSUM = enUmeration of Mortality and morbidity, P-POSSUM = Portsmouth POSSUM, CR-POSSUM = colorectal POSSUM.

The result of ROC analysis showed that all the scoring systems had good discrimination ability and all the values of AUC were more than 0.7 (Fig. 1). The Hosmer–Lemeshow test revealed that all the scoring system fit the data well, except the ACPGBI system (Table 2). However, these systems over-predicted the mortality rate according to the observed/predicted ratio (O/E).

**Table 2:**
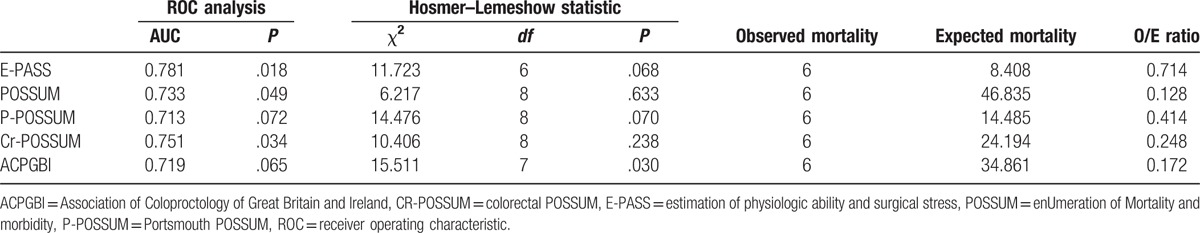
Efficiency of POSSUM-based system, ACPGBI system, and E-PASS system in predicting postoperative mortality.

### 3.3 Postoperative Complications

Among all 550 cases, 143 (26.00%) patients developed postoperative complications and 5 of them underwent reoperation. The postoperative morbidity rate of open surgeries and laparoscopic surgeries were 28.46% and 19.61%, which was significant higher in open surgeries (Z = −2.449, *P* = .012). In total 129 cases developed Grade II complication, 4 developed grade III complication, and 10 developed grade IV complication. Difference between open surgery and laparoscopic surgery were only found in Grade II complication (χ^2^ = 4.929, *P* = .026).

The result of univariate analysis demonstrated that there were significant differences between those with and without complications in terms of age, performance status, history of hypertension, diabetes mellitus, preoperative ileus, white blood cell, albumin, ASA grade, emergency surgery, laparoscopic surgery, radical resection and peritoneal soiling. Among factors above, white blood cell count, preoperative ileus, grade2&3 hypertension, diabetes mellitus, emergency surgery, and ASA grade III&IV were independent risk factors for postoperative complications according to multivariate analysis (Table 3).

**Table 3:**
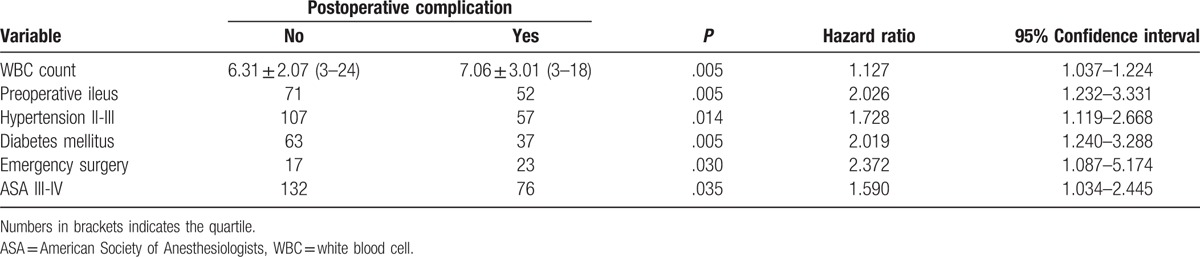
Logistic regression analysis of factors to postoperative complications.

POSSUM is the only system that has an explicit formula to predict postoperative morbidity among the systems aforementioned, but it did not show good discrimination ability with the AUC of 0.617.

### 3.4 Difference of postoperative morbidity between laparoscopic and open colorectal cancer surgery in patients with the different physiological risk

**Figure 2:**
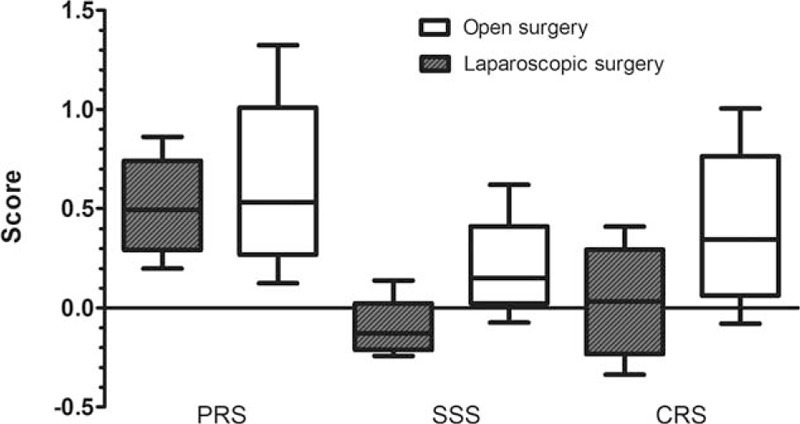
Differences of PRS, SSS, and CRS between open surgery and laparoscopic surgery. The PRS, SSS, and CRS are significantly lower in laparoscopic surgery. *Z* values were –2.895, –17.741, and –13.548. The *P* values were .004, .000, and .000. CRS = comprehensive risk score, PRS = preoperative risk score, SSS = surgical risk score.

There were significant differences of PRS, SSS, and CRS between laparoscopic and open surgery (Fig. 2), which meant that both physiological and surgical risks of laparoscopic surgery were lower than open surgery in 550 reviewed cases. The correlation analysis showed that the PRS, SSS, and CRS had positive correlation with postoperative morbidity. The coefficient of association were 0.185 (*P* = .000), 0.151 (*P* = .000), and 0.220 (*P* = .000).

**Figure 3:**
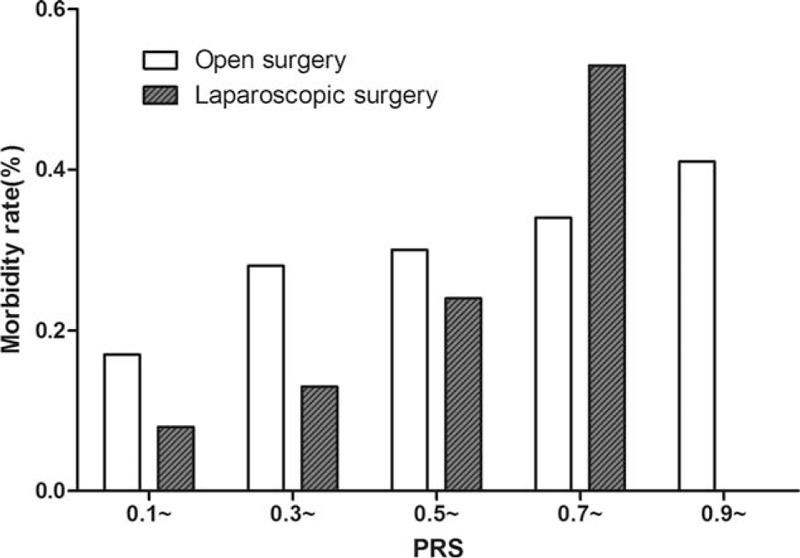
Variation trend of postoperative morbidity rate in open surgery and laparoscopic surgery. PRS of all cases were more than 0.1. There was no case of laparoscopic surgery when PRS is over 0.9. PRS = preoperative risk score.

On the whole, the postoperative morbidity rate of open surgeries is significantly higher than laparoscopic surgery (χ^2^ = 6.254, *P* = .012). But the differences would be inconsistent in different intervals of PRS. Statistical details and variation trend was demonstrated in Table 4 and Fig. 3. According to our results, the PRS value of 0.7 was the watershed for 2 surgical methods in terms of postoperative morbidity. When PRS was more than 0.7, the postoperative morbidity rate of laparoscopic surgeries would be higher than open surgeries.

**Table 4:**
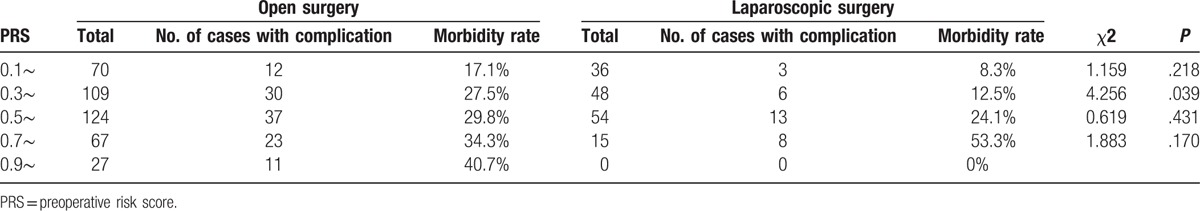
Postoperative morbidity rate of open and laparoscopic surgeries in different PRS intervals.

## 4 Discussion

Surgery is an important treatment for resectable colorectal cancer. As a traumatic procedure, colorectal surgeries have a certain extent of risk. So researchers found out factors that influence the surgical risk by analyzing large amount of cases and developed different scoring system to assess the surgical risk more objectively. In our study, we reviewed E-PASS, POSSUM, P-POSSUM, Cr-POSSUM, and ACPGBI in 550 colorectal cancer surgery cases. Result showed that all 5 scoring systems had good discrimination and calibration power, but on the other hand, they all over-predicted the mortality. In 5 scoring systems, E-PASS seemed to be the most accurate predicting system with the highest AUC, and the O/E value which was closest to 1. Compared to POSSUM-based systems, E-PASS used lesser scoring factors to assess the physiological status and surgical risk, which made E-PASS much easier in clinical use. However, the SSS score in the E-PASS system still utilizes the factors which can only be obtained postoperatively, such as operation time and blood loss. Technically, these scoring systems including E-PASS can only “predict” death after operation. Haga et al, therefore, developed modified E-PASS (mE-PASS) which assess surgical risk according to the type of surgical procedure rather than the concrete factors during the operation.^[19]^ The mE-PASS showed a similar predicting ability with E-PASS in some researches,^[12,13]^ but it also had limitations in clinical use. In the mE-PASS system, only routine surgical procedure can be assessed, whereas procedures such as emergency surgery or multiple organ resections cannot be assessed.

E-PASS has no designed formulas to assess the risk of postoperative morbidity, but the CRS score of E-PASS system was reported to have correlation with postoperative morbidity.^[20,21]^ In another research, PRS and CRS showed good discrimination ability on detecting postoperative complications with AUC values more than 0.7.^[22]^ So we further analyzed the relationship of PRS, SSS, and CRS with postoperative morbidity and their characteristics in 2 groups. The result of Spearman correlation tests showed that PRS, SSS, and CRS had a weak positive correlation with postoperative morbidity. Moreover, the laparoscopic surgery group had lesser PRS, SSS, CRS score, and lower morbidity rate than the open surgery group. The difference in PRS indicated that surgeons in our center tended to perform laparoscopic surgery for patient who had a relatively good physiological status. The result of SSS between 2 groups was as expected, since the open surgery has an excessively more weighting in SSS calculating formula.

Minimal invasive surgery is the general tendency for surgical evolution. Laparoscope is a typical minimal invasive technique and has been applied in various surgeries. As the laparoscopic technique gradually became mature, there were few absolute contraindications for laparoscopic colorectal surgery. T4 tumors and aged patients which used to be controversial for laparoscopic colorectomy were proved safe by some reports.^[17,23]^ Contraindications such as evident intestinal tympanites, mass ascites, and severe cardiopulmonary dysfunction were also very risky for open surgery. Numerous researches had been carried out to verify the safety and effectiveness of laparoscopic surgeries, and most of them gave us promising results.^[16,24]^ A study on 121,910 patients who underwent colorectal surgery concluded that laparoscopic colorectal surgery had advantage on postoperative morbidity rate, mortality rate, hospital costs, and hospital stays over open surgery.^[24]^ The difference between open surgery and laparoscopic surgery on the postoperative complication rate in our study was consistent with other reports. These optimistic results seemed to point out that laparoscopic colorectal cancer surgeries were fully superior to open surgeries. Current studies were seldom focused on the outcome of laparoscopic surgery for high risk patients. Most of these studies used age as the main sectionalization parameter. The result of a study on 408 rectal cancer patients demonstrated that laparoscopic surgery had clinical advantage over open surgery in patients under 80 years, but this advantage would be lost in patients over 80 years.^[17]^

The physiological status of patients is intricate, so that it is difficult to evaluate comprehensively and objectively. Age cannot comprehensively evaluate the physiological status of patients, even though it has a great impact on physiological reserve. By contrast, scoring systems include influencing factors comprehensively and values such as PRS can be an appropriate parameter to evaluate the physiological status of patients. According to the result of univariate and multivariate analysis, 6 factors that constitute the PRS score included 2 influencing factors and 2 independent influencing factors, whereas SSS only included 1 factor of open surgery. The value of PRS can be obtained preoperatively; therefore, it is possible for surgeons to evaluate physiological status of patients objectively and make appropriate decisions on surgical procedures based on the PRS value. So we analyzed the differences between the laparoscopic surgery group and the open surgery group in different intervals of PRS. Even though statistic differences were significant in only 1 interval (0.3–0.5), an evident trend still could be illustrated by Fig. 2. When PRS was less than 0.7, the morbidity rate of open surgery group was higher, but the gap is gradually narrowed as the PRS increases. When PRS was more than 0.7, the morbidity rate of laparoscopic surgeries became higher abruptly and exceeded the morbidity rate of open surgeries, though the difference is not significant. In 153 patients who underwent laparoscopic surgeries, maximum PRS was 0.862, so there was only open surgery cases in the last PRS interval (>0.9). It may be because the surgeons would like to perform open surgery for patients with high physiological risk, which could reduce surgery and anesthesia duration to mitigate the surgical risk.

It was a pity that we only included 550 cases and were unable to compare the morbidity rate in patients with high physiological risk. Further analysis on a larger sample is needed to verify present results. Other potential problems could be subjective factors such as the skill level of surgeons. In our center, colorectal cancer surgeries were performed by surgeons from the general surgery department. According to the report of Ferjani et al,^[25]^ colorectal surgeries performed by colorectal specialists had the lower mortality rate than other surgeons. Prystowsky et al^[26]^ also concluded that the surgeon would be the influencing factor of colorectal surgery outcome. Joint researches with other centers may be a solution.

In conclusion, E-PASS is an efficient scoring system for colorectal cancer surgery. The PRS value in E-PASS can be used as advice for surgeons in selecting surgical approaches for colorectal cancer surgery. Laparoscopic surgery could be better option for patients with a PRS value less than 0.7, whereas conventional open surgery would be safer for high physiological risk patients with a PRS value more than 0.7.

## References

[1] Kim NK Kim YW Han YD Complete mesocolic excision and central vascular ligation for colon cancer: Principle, anatomy, surgical technique, and outcomes. Surg Oncol 2016;25:252–62. 27566031 10.1016/j.suronc.2016.05.009

[2] van Gijn W Marijnen CAM Nagtegaal ID Preoperative radiotherapy combined with total mesorectal excision for resectable rectal cancer: 12-year follow-up of the multicentre, randomised controlled TME trial. The Lancet Oncology 2011;12:575–82. 21596621 10.1016/S1470-2045(11)70097-3

[3] Baer C Menon R Bastawrous S Emergency presentations of colorectal cancer. Surg Clin N Am 2017;97:529–45. 28501245 10.1016/j.suc.2017.01.004

[4] Copeland GP Jones D Walters M POSSUM: a scoring system for surgical audit. Brit J Surg 1991;78:355–60. 2021856 10.1002/bjs.1800780327

[5] Prytherch DR Whiteley MS Higgins B POSSUM and Portsmouth POSSUM for predicting mortality. Physiological and Operative Severity Score for the enUmeration of Mortality and morbidity. Brit J Surg 1998;85:1217–20. 9752863 10.1046/j.1365-2168.1998.00840.x

[6] Tekkis PP Poloniecki JD Thompson MR Operative mortality in colorectal cancer: prospective national study. BMJ 2003;327:1196–201. 14630754 10.1136/bmj.327.7425.1196 PMC274053

[7] Tekkis PP Prytherch DR Kocher HM Development of a dedicated risk-adjustment scoring system for colorectal surgery (colorectal POSSUM). Brit J Surg 2004;91:1174–82. 15449270 10.1002/bjs.4430

[8] Yan J Wang YX Li ZP Predictive value of the POSSUM, p-POSSUM, cr-POSSUM, APACHE II and ACPGBI scoring systems in colorectal cancer resection. J Int Med Res 2011;39:1464–73. 21986149 10.1177/147323001103900435

[9] Law WL Lam CM Lee YM Evaluation of outcome of laparoscopic colorectal resection with POSSUM, Portsmouth POSSUM and colorectal POSSUM. Brit J Surg 2006;93:94–9. 16288451 10.1002/bjs.5183

[10] Haga Y Wada Y Takeuchi H Estimation of physiologic ability and surgical stress (E-PASS) for a surgical audit in elective digestive surgery. Surgery 2004;135:586–94. 15179364 10.1016/j.surg.2003.11.012

[11] Haga Y Miyamoto A Wada Y Value of E-PASS models for predicting postoperative morbidity and mortality in resection of perihilar cholangiocarcinoma and gallbladder carcinoma. HPB 2016;18:271–8. 27017167 10.1016/j.hpb.2015.09.001 PMC4814599

[12] Haga Y Wada Y Takeuchi H Evaluation of modified estimation of physiologic ability and surgical stress in gastric carcinoma surgery. Gastric Cancer 2012;15:7–14. 21538017 10.1007/s10120-011-0052-2

[13] Haga Y Wada Y Ikenaga M Evaluation of modified estimation of physiologic ability and surgical stress in colorectal carcinoma surgery. Dis Colon Rectum 2011;54:1293–300. 21904145 10.1097/DCR.0b013e3182271a54

[14] Clinical Outcomes of Surgical Therapy Study G. A comparison of laparoscopically assisted and open colectomy for colon cancer. N Engl J Med 2004;350:2050–9. 15141043 10.1056/NEJMoa032651

[15] Lujan J Valero G Biondo S Laparoscopic versus open surgery for rectal cancer: results of a prospective multicentre analysis of 4,970 patients. Surg Endosc 2013;27:295–302. 22736289 10.1007/s00464-012-2444-8

[16] Schiphorst AH Verweij NM Pronk A Non-surgical complications after laparoscopic and open surgery for colorectal cancer - A systematic review of randomised controlled trials. Eur J Surg Oncol 2015;41:1118–27. 25980746 10.1016/j.ejso.2015.04.007

[17] Landi F Vallribera F Rivera JP Morbidity after laparoscopic and open rectal cancer surgery: a comparative analysis of morbidity in octogenarians and younger patients. Colorectal Dis 2016;18:459–67. 26408287 10.1111/codi.13136

[18] Dindo D Demartines N Clavien P-A Classification of surgical complications. Ann Surg 2004;240:205–13. 15273542 10.1097/01.sla.0000133083.54934.ae PMC1360123

[19] Haga Y Ikejiri K Wada Y A multicenter prospective study of surgical audit systems. Ann Surg 2011;253:194–201. 21233616 10.1097/SLA.0b013e3181f66199

[20] Haga Y Ikei S Ogawa M Estimation of Physiologic Ability and Surgical Stress (E-PASS) as a new prediction scoring system for postoperative morbidity and mortality following elective gastrointestinal surgery. Surg Today 1999;29:219–25. 10192731 10.1007/BF02483010

[21] Haga Y Wada Y Takeuchi H Prediction of anastomotic leak and its prognosis in digestive surgery. World J Surg 2011;35:716–22. 21184072 10.1007/s00268-010-0922-5

[22] Tominaga T Takeshita H Takagi K E-PASS score as a useful predictor of postoperative complications and mortality after colorectal surgery in elderly patients. Int J Colorectal Dis 2016;31:217–25. 26607908 10.1007/s00384-015-2456-7

[23] Shukla PJ Trencheva K Merchant C Laparoscopic resection of t4 colon cancers: is it feasible? Dis Colon Rect 2015;58:25–31. 10.1097/DCR.0000000000000220 25489691

[24] Kang CY Chaudhry OO Halabi WJ Outcomes of laparoscopic colorectal surgery: data from the Nationwide Inpatient Sample 2009. Am J Surg 2012;204:952–7. 23122910 10.1016/j.amjsurg.2012.07.031

[25] Ferjani AM Griffin D Stallard N A newly devised scoring system for prediction of mortality in patients with colorectal cancer: a prospective study. Lancet Oncol 2007;8:317–22. 17395105 10.1016/S1470-2045(07)70045-1

[26] Prystowsky JB Bordage G Feinglass JM Patient outcomes for segmental colon resection according to surgeon's training, certification, and experience. Surgery 2002;132:663–72. 12407351 10.1067/msy.2002.127550

